# Giant pelvic abscess with sepsis: Case report and review of current literature

**DOI:** 10.1016/j.ijscr.2019.10.002

**Published:** 2019-10-07

**Authors:** Adel Elkbuli, Kyle Kinslow, Brandon Diaz, Shaikh Hai, Mark McKenney, Dessy Boneva

**Affiliations:** a Department of Surgery, Kendall Regional Medical Center, Miami, FL, USA; b University of South Florida, Tampa, FL, USA

**Keywords:** Pelvic abscess, Sepsis, Surgical drainage, Surgical outcomes

## Abstract

**Introduction:**

Intra-abdominal and pelvic abscesses can develop because of multiple etiologies. Typically, these abscesses are managed via a combination of medical (antibiotics) and surgical (drainage) interventions. This is a unique case report of a giant idiopathic pelvic abscess with necrotizing fasciitis that failed conservative management with antibiotics, requiring urgent surgical intervention and complex closure of the wound.

**Case presentation:**

A 71-year-old female presented to the emergency department in sepsis, complaining of right hip and flank pain of one-month duration. Imaging revealed a giant right flank abscess with necrotizing fasciitis. The abscess extended from the pelvis to the right retroperitoneal space, traversing several soft tissue planes, to the skin. She was treated emergently with open surgical drainage, debridement, and delayed complex closure. The patient significantly improved post-operatively and was subsequently discharged.

**Discussion:**

Pelvic abscesses are commonly due to gastrointestinal, genitourinary, post-surgical, or rarely idiopathic causes. US, CT, or MRI are the imaging modalities effective in diagnosing and staging abscess severity. Currently percutaneous, image-guided abscess drainage is the primary management due to it being minimally invasive, having better outcomes and reduced hospital stay. In large, multi-loculated abscess as exhibited by our patient, open surgical drainage and debridement of the infected cavity was not only successful but imperative in effective resolution.

**Conclusion:**

Most pelvic abscesses can be treated with percutaneous drainage but those complicated by necrotizing fasciitis, or multi-loculated collections may be better treated with open drainage.

## Highlights

• We present a case of massive pelvic abscess complicated by sepsis and necrotizing fasciitis successfully treated with surgical drainage.
• There was complete resolution of sepsis following open surgical drainage of a massive pelvic abscess complicated by necrotizing fasciitis.
• Larger multi-loculated abscesses or when associated with necrotizing infections may be better approached surgically.

## 1 Introduction

An abscess is a defined collection of purulent fluid that has accumulated within the tissues or cavities of the body; this is often a result of an infectious process. They can be located in different areas of the body such as the skin, intra-abdominal, pelvic, and retroperitoneum. Intra-abdominal and pelvic abscesses can develop because of multiple etiologies including complications of surgery or from infectious processes such as inflammatory bowel disease, diverticulitis, or pelvic inflammatory disease. Specifically, pelvic abscesses can develop as a complication of gynecologic surgeries such as hysterectomy and cesarean section [1]. It is estimated that fewer than 1% of patients that had obstetric or gynecologic surgery develop an abscess [2]. Pelvic abscesses in particular can be challenging to drain surgically due to anatomy and inflammation [3].

Typically, abscesses are managed via a combination of medical (antibiotics) and surgical (drainage) interventions. Antibiotics are initiated, the collection is typically drained, fluid sent for culture and antibiotics adjusted based on culture results. Medical management alone with antibiotics can be sufficient for collections of small caliber, or those that are not yet well organized. Drainage of abscesses is determined by symptomology, clinical status of the patient, size and location of abscess, and failed treatment that is more conservative. Drainage of intra-abdominal, visceral, and pelvic abscesses, can be accomplished with percutaneous drainage instead of a more invasive surgical drainage; however, surgical abscess drainage is indicated for hemodynamically unstable patients and those who have failed more conservative measures (antibiotics and/or percutaneous drainage), or for multi-loculated abscesses. This work has been reported in line with the SCARE criteria [4].

## 2 Case presentation

A 71-year-old female presented to the emergency department complaining of right hip and flank pain that began approximately 1 month prior to arrival. Of note, she had a hysterectomy 2 years prior that was uneventful. She also stated that 15 days prior she noted redness and swelling in the same area where the pain is located. The area began to spontaneously drain purulent material 2 days prior to her presentation. Because of her ongoing pain she had previously visited her primary physician and an urgent care, and on both occasions had been prescribed oral antibiotics, which did not improve her symptoms. She denied fever, chills, headaches, dizziness, abdominal pain, nausea, vomiting, dysuria or any other symptoms. She also denied any trauma to the area.

**Fig. 1:**
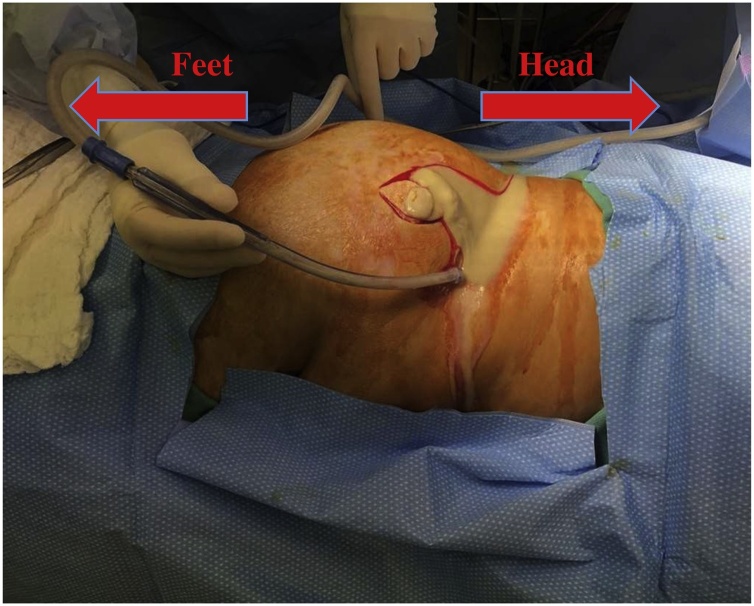
Large, indurated mass on the right hip/flank spontaneously draining purulent fluid (patient is positioned in left lateral decubitus).

**Fig. 2:**
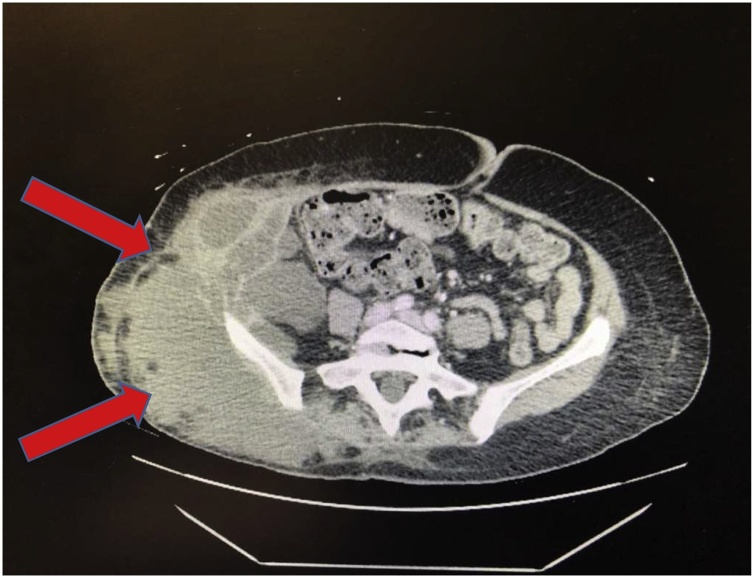
Trans spatial, multiloculated fluid collection containing gas bubbles, extending through the right abdominal side wall involving the retroperitoneal space, abdominal/pelvic side wall musculature, and in the subcutaneous fat compatible with abscess.

Upon physical exam there was found to be a large, indurated, fluctuant mass on the right hip/flank which was spontaneously draining pus from a small opening (Fig. 1). She was diagnosed with sepsis because of her tachycardia, tachypnea, thrombocytosis, and marked leukocytosis. Computed tomography (CT) demonstrated a trans-fascial, multi-loculated fluid collection containing gas bubbles, extending through the right abdominal sidewall involving the retroperitoneal space, abdominal/pelvic side wall musculature, and in the subcutaneous fat compatible with abscess (Fig. 2). The CT scan did not reveal any intra-peritoneal pathology; specifically no colonic diverticulosis, no bowel wall thickening, no reactive mesenteric infiltration or free fluid to suggest an enteric source of this suppurative process. We could not identify any post-hysterectomy residual chronic adnexal inflammation either.

**Fig. 3:**
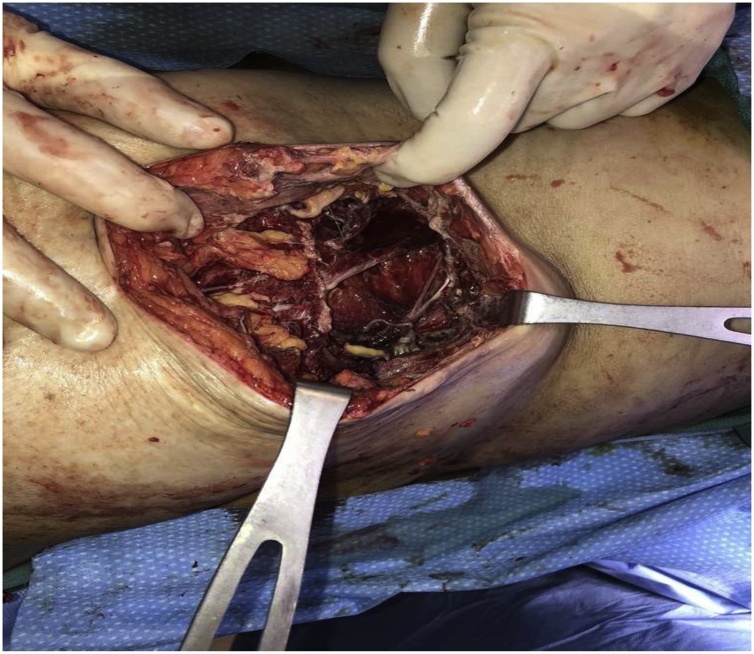
Wound defect after extensive debridement measured to be 19 cm × 20 cm × 20 cm. Clear muscle and soft tissue involvement noted.

**Fig. 4:**
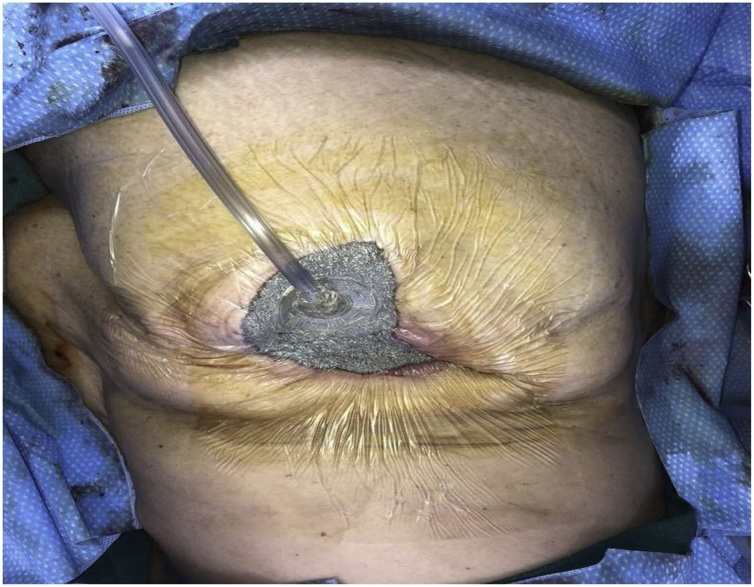
Wound VAC applied to wound defect after drainage and debridement to prepare the wound for second look and closure.

Due to the patient’s clinical picture and the concern for necrotizing soft tissue infection the patient was taken emergently to the operating room for drainage, washout, and debridement. A giant abscess was encountered with 3 liters of purulent fluid; intraoperative cultures were taken and sent to microbiology to identify causative organism(s). Following drainage, extensive excisional debridement was undertaken, until all necrotic tissue was removed. The wound defect was measured to be 19 cm × 20 cm × 20 cm (Fig. 3). Due to the large defect and grossly contaminated operative field, a negative pressure wound dressing, vacuum assisted closure dressing (VAC) was applied to prepare the wound for a secondary examination and delayed primary closure (Fig. 4).

Immediately post-operatively the patient had significant relief of her symptoms, her vital signs normalized, and her white blood cell count began to trend down. She was taken back to the operating room 3 days later for a second look. No further wound debridement was needed. Delayed primary wound closure was accomplished. Two Jackson-Pratt drains were placed in the cavity for further drainage. According to the culture results, microbiology was not able to identify a particular predominant organism, however multiple gram-positive rods were noted and there were no anaerobes seen. She further improved and was discharged 3 days later (hospital day 6) on oral antibiotics (as recommended by infectious disease specialist), and home health for drain management. She was seen in the clinic 10 days after discharge, where the sutures and drains were removed. There were no post-operative complications noted at this time; the incision was healing well and there was no sign of infection. She was seen again 2 weeks later in clinic and no post-operative complications were noted. She was referred to a gastroenterologist to perform an elective interval colonoscopy as well.

## 3 Discussion

Typically, pelvic abscesses in women form secondary to underlying genitourinary pathology mainly due to pelvic inflammatory disease (tubo-ovarian abscess or salpingitis). Other common causes in both men and women include but are not limited to gastrointestinal manifestations (complicated diverticulitis, bowel perforation, appendicitis, inflammatory bowel disease such as Crohn’s disease), bacterial seeding from local trauma or prior surgery, or idiopathic etiology. When considering management of pelvic abscesses, multiple imaging modalities are available for assessment of abscess characteristics such as size, constitution, and local anatomy impacted by this space occupying lesion. Ultrasonography is the most rapid, least-invasive modality and can be performed via transabdominal, transvaginal, transrectal, or transgluteal routes depending on the patient’s clinical presentation and regional symptomatology. When a patient presents with nonspecific symptoms of abdominal pain with no clear underlying cause or if there is concern for abscess sequelae, MRI or CT imaging is very useful in detecting an underlying abscess and better understanding its intrinsic characteristics and local impact [5,6]. In our presented case, the patient presented with obvious physical deformity of the right flank/hip due to the massive size of the abscess itself. The spontaneous drainage of the abscess in conjunction with the clinical signs of systemic inflammatory reaction made the underlying pathology clear, however, CT was used in particular to assess its extent of local tissue involvement, as well as identify any possible primary pathology causing such a massive abscess to form.

Historically, laparotomy with lavage or surgical incision with drainage was the mainstay of pelvic abscess treatment. However, current approaches to pelvic abscess management often involve ultrasound and CT guided percutaneous drainage of both deep organ-space and superficial abscesses which has shown significant success in abscess elimination, do not require general anesthesia, and are ultimately less invasive so long that the abscess is not complicated by compartmentalization or fistulation [7]. Compared to laparoscopy or surgical drainage, evidence exists suggesting lower hospital stay as well as lower rates of surgical complications [8]. These results are supported by a recent retrospective review conducted by Akinci et al., in which 185 pelvic abscesses of multiple etiologies were managed via image-guided drainage to assess overall success of the approach. Of note, the median volume drained amongst these cases was 147 ml with the largest being 780 ml. Furthermore, the patient population involved in the success of their approach mainly consisted of smaller, contained abscesses without significant extension/impact on local tissues. Despite such evidence, open surgical drainage and debridement was decided for our patient due to the massive size of the abscess (ultimately total of 3 liters drained) in addition to the presence of sepsis and signs of necrotizing fasciitis on CT imaging with distant involvement. A less invasive approach such as an image-guided drainage would have likely been insufficient in containing the extensive multi-loculated necrotic tissue infection or resolving the clinical sepsis exhibited by our patient. However, there is even debate on whether image-guided drainage is inferior to surgical approach despite such complications [9].

By far the most impressive aspect of our case was the abscess size on presentation and final wound size (19 cm × 20 cm × 20 cm) despite no clear underlying pathology that may have led to its inception. This case is, to our knowledge, one of the largest pelvic abscesses to be successfully treated and documented in the literature. Our patient’s only relevant history was a total hysterectomy performed two years prior with her recovery course being uncomplicated, without infection or post-operative abscess formation. It is apparent that while idiopathic pelvic abscesses may constitute a minority of pelvic abscess cases, nonetheless they do have potential for massive growth and systemic infection dissemination [7]. Our patient was initially treated conservatively with pain management and antibiotic therapy which did not prevent her abscess from progressing to necrotizing infection and sepsis. While the current trend now leans toward utilization of less invasive percutaneous drainage methods for pelvic abscess management (possibly for even severe cases), our case shows that emergent open surgical management of such a remarkably large very thick abscess showing extensive dissemination was not only sufficient in resolution of symptoms but also did not result in any perioperative complications as a result of the surgical approach and was ultimately lifesaving. Additionally, the expediency of our surgical intervention, knowing well the clinical status of the patient, size of the abscess, and high suspicion for necrotizing fasciitis, was instrumental in the good outcome of our case. Open surgical drainage should continue to remain a valuable consideration in the management of severe, emergent pelvic abscess infections especially when percutaneous drainage is suspected to fail and prolong the clinical course.

## 4 Conclusion

We present the case of a 71 year old female patient with a massive pelvic/flank abscess complicated by sepsis and concomitant necrotizing fasciitis preferentially treated successfully with antibiotics, emergent surgical drainage, and open wound debridement. Most pelvic abscesses can be successfully treated with percutaneous drainage but multi-loculated, giant abscesses or abscesses complicated by transfascial extension or necrotizing infections are better treated with open surgical drainage and debridement.

## Sources of funding

None.

## Ethical approval

This report was conducted in compliance with ethical standards. Informed written consent has been obtained and all identifying information is omitted.

## Consent

Informed consent has been obtained and all identifying information is omitted.

## Author contributions

Adel Elkbuli, Dessy Boneva, Brandon Diaz - Conception of study, acquisition of data, analysis and interpretation of data.

Adel Elkbuli, Dessy Boneva, Kyle Kinslow, Brandon Diaz, Mark McKenney - Drafting the article.

Dessy Boneva, Mark McKenney - Management of case.

Adel Elkbuli, Brandon Diaz, Kyle Kinslow, Shaikh Hai, Dessy Boneva, Mark McKenney - Critical revision of article and final approval of the version to be submitted.

## Registration of research studies

This is a case report study.

## Guarantor

Dessy Boneva.

Mark McKenney.

## Provenance and peer review

Not commissioned, externally peer-reviewed.

## Declaration of Competing Interest

None.

## References

[1] Berríos-Torres S.I., Umcheid C.A., Bratzler D.W. (2017). Centers for disease control and prevention guideline for the prevention of surgical site infection, 2017. JAMA Surg..

[2] Mahdi H., Goodrich S., Lockhart D., DeBernardo R., Moslemi-Kebria M. (2014). Predictors of surgical site infection in women undergoing hysterectomy for benign gynecologic disease: a multicenter analysis using the national surgical quality improvement program data. J. Minim. Invasive Gynecol..

[3] Chappell C.A., Wiesenfeld H.C. (2012). Pathogenesis, diagnosis, and management of severe pelvic inflammatory disease and tuboovarian abscess. Clin. Obstet. Gynecol..

[4] Agha R.A., Borrelli M.R., Farwana R. (2018). The SCARE 2018 statement: updating consensus Surgical CAse REport (SCARE) guidelines. Int. J. Surg..

[5] Granberg S., Gjelland K., Ekerhovd E. (2009). The management of pelvic abscess. Best Pract. Res. Clin. Obstet. Gynaecol..

[6] Robert B., Chivot C., Fuks D., Gondry-Jouet C., Regimbeau J.M., Yzet T. (2013). Percutaneous, computed tomography-guided drainage of deep pelvic abscesses via a transgluteal approach: a report on 30 cases and a review of the literature. Abdom. Imaging.

[7] Akıncı D., Ergun O., Topel Ç, Çiftçi T., Akhan O. (2018). Pelvic abscess drainage: outcome with factors affecting the clinical success. Diagn. Interv. Radiol..

[8] Khaliq K., Nama N., Mahdy H. (2019). Pelvic abscess [Updated 5 August 2019]. StatPearls [Internet].

[9] Schein M. (1970). Management of intra-abdominal abscesses. Surgical Treatment: Evidence-Based and Problem-Oriented.

